# Hepatitis C virus infection and its associated factors among prisoners in a Nigerian prison

**DOI:** 10.1186/s12876-020-01504-8

**Published:** 2020-10-30

**Authors:** Ifeyinwa M. Okafor, Solomon O. Ugwu, Henshaw U. Okoroiwu

**Affiliations:** Haematology Unit, Department of Medical Laboratory Science, College of Medical Sciences, University of Calabar, Calabar, Nigeria

**Keywords:** Hepatitis C, Prison, Infectious disease, Inmates, Hepatitis C prevalence

## Abstract

**Background:**

The prison population is considered at high risk of acquiring infectious diseases due to confined conditions, behavioral factors, injection drug use, unprotected sexual activity, non-professional tattooing and scarification, and needle sharing. Hepatitis C virus (HCV) is a blood borne pathogen mostly transmitted via percutaneous exposure that results in inflammation of the liver. It is one of the public health problem worldwide and is the principal cause of parenterally transmitted non-A, non-B hepatitis. The study was aimed at evaluating the prevalence of HCV among prison inmates in Calabar, Cross River State, Nigeria and the associated factors.

**Methods:**

The study took a descriptive cross sectional approach using multi-stage sampling technique. One hundred and forty-two (142) prison inmates within the age range of 18–50 years and above were recruited for this study.

**Result:**

Forty two (42); [29.6%] of the participating prison inmates were seropositive for HCV. Gender stratification showed that 31.0% of the males were seropositive for HCV while 15.4% of the females were seropositive for HCV. Fisher exact test showed that gender, age, marital status, occupation and level of education had no association in distribution of seroprevalence of HCV (*p* > 0.05) but the duration in prison was significantly associated with distribution of seropositivity of HCV in the studied population (*p* < 0.05). Bivariate logistic regression showed that tattoo/scarification, injection drug use, history of blood transfusion, sexual experience, shaving equipment sharing and multiple sexual partners were not risk factor for distribution of HCV prevalence in the studied population (*p* > 0.05). However, 23.5% who had tattoo/scarification, 29.6% who used injection drug, 33.3% who had history of blood transfusion, 29.8% who had sexual experience, 21.2% who shared shaving equipment, and 28.3% who had multiple sex partners were seropositive for HCV.

**Conclusion:**

Approximately 29.6% prevalence of Hepatitis C virus infection observed among inmates studied is high and calls for concern. Attitude and behaviors by inmates such as tattooing/scarification, injection drugs use, sharing of shaving equipment, multiple sexual partners should be discouraged.

## Background

Hepatitis C virus (HCV) is a blood borne pathogen mostly transmitted via percutaneous exposure [1] that results in inflammation of the liver and is linked to a variety of adverse health outcomes including hepatocellular cancer and cirrhosis [2, 3]. It is a global public health problem and is the principal cause of parenterally transmitted non-A, non-B hepatitis [4, 5]. The 2015 global prevalence of HCV as reported by WHO (2017) estimated that 71 million persons were living with HCV infection, accounting for 1% of global population [6, 7]. Regional stratification showed highest prevalence in the Eastern Mediterranean region (2.3%) and European region (1.5%), while African region, Region of Americas, Western Pacific region and South East Asia regions had 1.0%, 0.7% and 0.5%, respectively. The prices of WHO—recommended direct acting antivirals (DAAs) for HCV vary substantially (US $200–$45,000) for a curative course [6].

Globally, there are more than 10.74 million people jailed in penal institutions either as pre-trial detainees/remand prisoners or having been convicted and sentenced [8]. Nigeria has the 5th highest number of prisoners (75,772) in Africa just below South Africa (164,129), Ethiopia (113,727), Egypt (106,000), and Morocco (82,512) [9] and 24th in the world [10] as of 2018. The United Nations basic principles for the treatment of prisoners state that “Prisoners shall have access to health services available in the country without discrimination on the grounds of their legal situation” [11]. However, in practice, this basic principle is scarcely applied in real life and prisoners in most countries have lesser possibility of medical assistance than the non-incarcerated citizens [12].

The prison population is considered at high risk of acquiring infectious diseases related to confined conditions owing to behavioral factors related to injection drug use, unprotected sexual activity, non-professional tattooing and scarification, and needle sharing [13]. Other risk factors such as surgery, intranasal cocaine use, previous blood transfusion have been documented [14–16]. However, there are contradictory reports on literature about the role of intrafamilial transmission of HCV via body contact and sharing of items [17]. More so, alcohol consumption has been associated with HCV infection disease progression [18], but not as a risk behavior. These risky life style may precede imprisonment and mostly continue during incarceration [19, 20]. Previous studies have shown that HCV infection is more frequently detected in prisoners than the general population [21–23].

The study was aimed at evaluating the prevalence of HCV among prison inmates in Calabar, Cross River State Nigeria and the associated factors (such as tattoo/scarification, injection drug use, history of blood transfusion, sexual experience, shaving equipment sharing, multiple sex partner and previous incarceration). The knowledge of the epidemiology will aid in designing and implementation of public health policy geared towards reducing prison “reservoir” of infectious diseases and possible transmission to fellow inmates and the society when released.

## Methods

### Study design

**Fig. 1:**
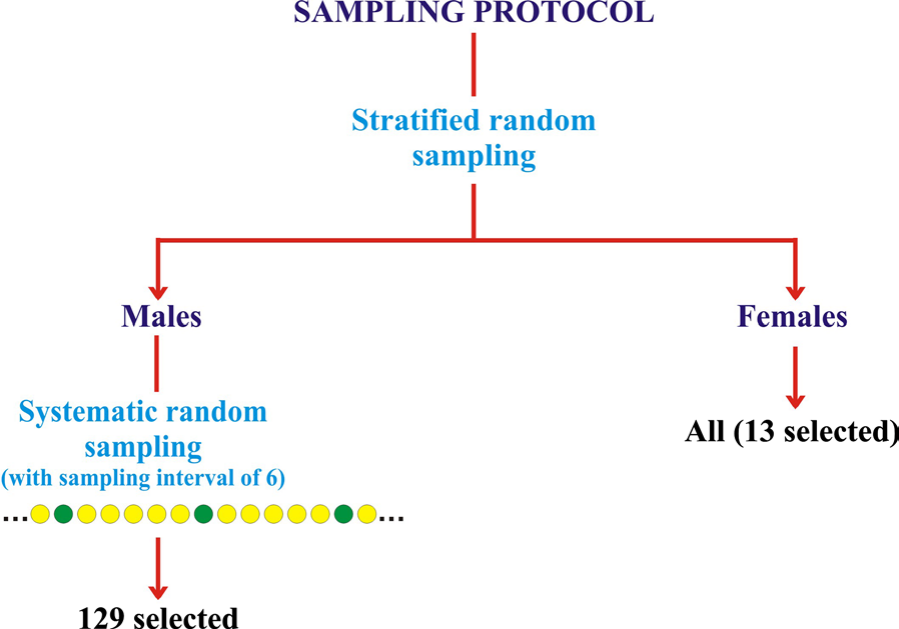
Flow diagram depicting process of multi-stage sampling of study participants

The study took a descriptive cross sectional approach using multi-stage sampling technique and was conducted from May 2018 to October 2018. On the day of the data collection, the prisoners were first stratified into male and female; all females were recruited (owing to the small number) while the males were numbered by random pick from folded papers inside a basket for systematic random sampling. The sampling interval was determined which is 6 and the starting number was determined by another random pick by the data collection team (Fig. 1).

### Study setting

**Fig. 2:**
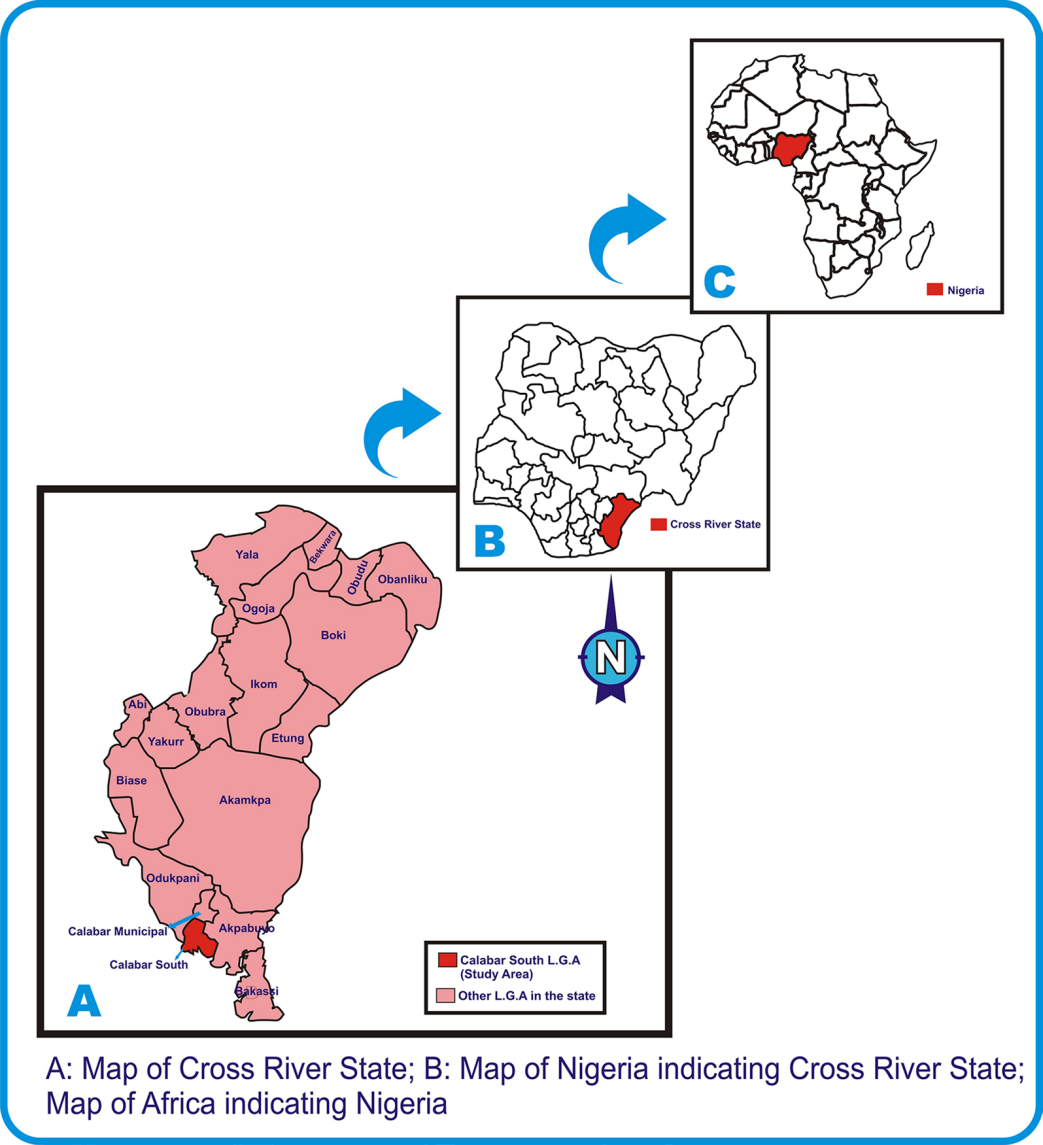
Map indicating the study setting [28]

The study was conducted in Calabar, Cross River State, Nigeria. Cross River is a state located in Southern Nigeria in the Niger Delta region [24]. It is bounded in the north by Benue State, the west by Ebonyi and Abia State, the east by Cameroon Republic and the south by Akwa Ibom and the Atlantic Ocean [25]. The State has an area of 21,787km^2^ and a population of 2,892,988 (2006 census) [26] distributed across 18 Local Government Areas (LGAs) [26, 27]. The prison is located in Afokang Street, Calabar South LGA. Calabar has a geographical coordinates of 4057′32.15"N, 8019′37.02"E with an estimated population of 375,196 (2006 census) [27] comprising of Calabar South and Calabar Municipality (Fig. 2) [28].

### Study population and sample size determination

The sample size was calculated based on expected HCV prevalence of 4.0% [29] with 5% alpha-type error (95 confidence interval.) as described by Naing 2003 [30]. Hence, the minimum sample size was 59 prisoners. Overall 142 prison inmates aged ≥ 18 years were randomly selected out of the 875 (male = 862: female = 13) serving for this study. We added 83 more individuals to account for anticipated drop-out or missing data while research is on and as well increase the power of the study. Hence, one hundred and forty two (142) prison inmates within the age range of 18 to 50 years and above were recruited for this study.

$$\begin{aligned} & n = \left( {\frac{z}{\Delta }} \right)^{2} p(1 - p) = z/\Delta^{2} p(1 - p) \\ & p = 0.04,\, \Delta = 0.05, \, z = 1.96 \\ & {1536}.{64}*0.0{4}*0.{96} \\ & {\text{n }} = { 59}.00{7} \\ \end{aligned}$$

Approximate minimum sample size n = 59.

where, n: required minimum sample size, z: standard normal variate of thr confidence level of 95% = 1.96, Δ: Desired precision/accuracy or margin of erro = 0.05, p: Estimated proportion in the population with the characteristics of interest from previous study = 4% = 0.04.

### Data collection

A structured questionnaire was shared to all participating prison inmates to obtain socio-demographic data after a prison warder introduced the researchers and the subsequent explanation of the research aim to the prisoners. The participating inmates were issued the questionnaire to fill themselves (as all were able to read and write) except in few cases where the inmates asked for clarifications in some questions and they entered the responses themselves. All filled questionnaires were collected in a big carton and shuffled. The questionnaire was structured to bear minimal identity as names were not included, rather, serial numbers were used to tally test results and subjects to rule out fear of stigmatization or possible punishment owing to answers provided by the inmates. The serial number on each questionnaire was duplicated on the sampling bottle for later coupling of result and subject.

### Inclusion and exclusion criteria

Subjects who were incarcerated and gave informed consent were recruited while subjects who were not incarcerated and incarcerated subjects who withheld consent were excluded.

### Specimen collection

Five milliliter (5 ml) of blood was collected from each subject and dispensed into sterile plain container and allowed to clot. The serum obtained was used for serological diagnosis of antibodies to hepatitis C virus.

### Assay procedure

The HCV assay was performed using serum immunochromatographic rapid diagnostic test (RDTs) for HCV (Anti-HCV) by ACON laboratories incorporated, USA. The serological protocol is a qualitative immunoassay detection of IgG antibodies of hepatitis C virus in the sample serum. The sensitivity and specificity of the test kits are 93% and 93%, respectively. The test procedure is antigen based. Serum sample was used for the assay.

### Statistical analysis

Data were analyzed using SPSS version 20 (Armonk, NY. IBM Corp). Categorical demographic and clinical variables were summarized in frequencies and Fisher exact test and Chi square was used to assess association between categorical variables, while bivariate logistic regression was used in evaluating risk factors. In the logistic regression, seropositivity/seronegativity of HCV was used as the dependent variable while the risk behaviors were used as independent variables. Significant difference was determined at *p* ≤ 0.05.

### Measures

The variables assessed in the study are gender, age (in years), marital status, level of education, occupation, duration in prison (in months), status of previous incarceration, presence of tattoo/scarification, previous blood transfusion, history of surgery, injection drug use, alcohol consumption, sexual experience, multiple sexual partners, history of STI, sharing of shaving equipment, sharing of needle and syringe and clothe sharing. The respective variables were extracted from the structured questionnaire. Multiple sex partner as a variable was defined as those who have had sexual intercourse with more than one partner within a 12 months or greater duration (Coded as one or less sexual partner versus more than one sexual partner). Variables such as; gender was stratified into male and female while; age was stratified into 18–27, 28–37, 38–47 and ≥ 48 years. The level of education of the subjects were stratified into primary, secondary, tertiary and non-formal while duration in prison was documented in months. Other variables such as risk behaviours and factors were assessed on yes and no status. The variables used in the regression model was based on previous reports of risk factors.

## Result

A total of 142 inmates participated in the study with male preponderance (90.8%, n = 129). Half of the studied population (50%, n = 71) were aged 18–27 years while those aged > 48 years constituted the least (4.2%, n = 6). Approximately, 39.4% (n = 56) of the studied subjects were married, while 56.3% (n = 80) were single. Majority of the inmates (53.5%, n = 76) had completed secondary school education. Most inmates were students (40.1%, n = 57) and businessmen (38.7%, n = 55). Forty two (29.6%) of the consenting prison inmates were seropositive for HCV. Gender stratification showed that 31.0% (n = 40/129) of the males were seropositive for HCV while 15.4% (n = 2/13) of the females were seropositive for HCV. Fisher exact test showed that gender had no association in distribution of seroprevalence of HCV in the studied population (*p* > 0.05). Stratification of the seroprevalence data by age showed the highest prevalence in the 28–37 years category (35.6%) followed by the ≥ 48 years category (33.3%). This order is followed by the 18–27 years (26.8%) and 38–47 years categories (25.0%). Age was found not to be associated with distribution of seroprevalence of HCV in the studied population (*p* > 0.05). Approximately 32.1% of the married, 28.8% single and 50.0% of the divorced subjects were seropositive for HCV. More so, 31.6% of the students, 50.0% civil servant, 30.8% of the public servants, 21.8% of the business persons and 46.2% of the unemployed inmates were seropositive for HCV. Assessment based on level of education showed that 50.0% of the primary, 25.0% of the secondary, 27.8% of the tertiary and 33.3% of the non-formal educated inmates were seropositive for HCV. Marital status, occupation and level of education were found not to be associated with seroprevalence for HCV in the studied population (*p* > 0.05). Overall, none of the socio-demographic variables was associated with HCV seroprevalence in the studied population (Table 1).

**Table 1:** Socio-demographic characteristics and assessment of their association with HCV infection among prison inmates in Calabar

Characteristics	Frequency (%)	No reactive (%)	*p* value
*Gender*			
Male	129 (90.8)	40 (31.0)	0.345*
Female	13 (9.2)	2 (15.4)	
*Age (years)*			
18–27	71 (50.0)	19 (26.8)	0.749*
28–37	45 (31.7)	16 (35.6)	
38–47	20 (14.1)	5 (25.0)	
≥ 48	6 (4.2)	2 (33.3)	
*Marital status*			
Married	56 (39.4)	18 (32.1)	0.860*
Single	80 (56.3)	23 (28.8)	
Divorced	2 (1.4)	1 (50.0)	
Separated	2 (1.4)	0 (0.0)	
Widowed	2 (1.4)	0 (0.0)	
*Level of education*			
Primary	22 (15.5)	11 (50.0)	0.140*
Secondary	76 (53.5)	19 (25.0)	
Tertiary	38 (26.8)	10 (27.8)	
Non-formal	6 (4.2)	2 (33.3)	
*Occupation*			
Student	57 (40.1)	18 (31.6)	0.323*
Public servant	17 (12.0)	6 (35.3)	
Business	55 (38.7)	12 (21.8)	
Unemployed	13 (9.2)	6 (46.2)	

^*^Fisher exact test

*No* number

In terms of HCV risk behaviors, 23.9% (n = 34) had tattoo/scarification, 10.6% (n = 15) had previous blood transfusion, 19.0% (n = 27) used injection drugs. In terms of other behaviors of relevance, 99.3% (n = 141) had sexual experience, 69.7% (n = 99) had multiple sexual partners, 22.5% (n = 32) had history of STI, 36.6% (n = 52) had shared shaving equipment, 9.2% (n = 13) had shared needle and syringe. In terms of other factors associated with HCV infection, 12.7% (n = 18) had history of surgery. More than half (59.9%, n = 85) had stayed in prison within 12 months, while 21.1% (n = 30) have stayed up to 2 years. Overall, 22 (15.5%) inmates had been previously incarcerated. Approximately 41.2% of the inmates who have been incarcerated within 12 months and 13.3% of those who have been incarcerated within 13–24 months were seropositive for HCV. Approximately 16.7% of those incarcerated within 25–26 months were seropositive for HCV. Fisher exact test showed that duration in prison was significantly associated with distribution of seropositivity of HCV in the studied population (*p* < 0.05). Approximately 36.4%, 23.5%, 33.3%, 38.9%, 29.6%, 29.8%, 28.3%, 25.0%, 21.2% and 38.5% of those who were previously incarcerated, had tattoo/scarification, had history of blood transfusion, had history of surgery, used injection drugs, had sexual experience, had multiple sex partners, had history of STI, shared shaving equipment and those who shared needle and syringes, respectively, were seropositive for HCV infection (Table 2).

**Table 2:** Risks associated with HCV infection among prison inmates in Calabar

Characteristics	Frequency (%)	No. positive (%)	X^2^	*P* value
*Duration in prison (months)*				
≤ 12	85 (59.9)	35 (41.2)		0.008*
13–24	30 (21.1)	4 (13.3)		
25–36	18 (12.7)	3 (16.7)		
37–48	3 (2.1)	0 (0.0)		
≥ 49	6 (4.2)	0 (0.0)		
*Previous incarceration*				
Yes	22 (15.5)	8 (36.4)	0.576	0.448
No	120 (84.5)	34 (28.3)		
*Presence of tatoo/scarification*				
Yes	34 (23.9)	8 (23.5)	0.785	0.376
No	108 (76.1)	34 (31.5)		
*Previous blood transfusion*				
Yes	15 (10.6)	5 (33.3)		0.768*
No	127 (89.4)	37 (29.1)		
*History of surgery*				
Yes	18 (12.7)	7 (38.9)	0.858	0.354
No	124 (87.3)	35 (28.2)		
*Injection drug use*				
Yes	27 (19.0)	8 (29.6)	0.000	1.000
No	115 (81.0)	34 (29.6)		
*Sexual experience*				
Yes	141 (99.3)	42 (29.8)		1.000*
No	1 (0.7)	0 (0.0)		
*Multiple sex partner*				
Yes	99 (69.7)	28 (28.3)	0.263	0.608
No	43 (30.3)	14 (32.6)		
*History of STI*				
Yes	32 (22.5)	8 (25.0)	0.416	0.519
No	110 (77.5)	34 (30.9)		
*Sharing of shaving equipment*				
Yes	52 (36.6)	11 (21.2)	2.795	0.095
No	90 (63.4)	31 (34.4)		
*Needle and syringe sharing*				
Yes	13 (9.2)	5 (38.5)		0.768
No	129 (90.8)	37 (28.7)		

All results are Chi square except where indicated with asterisk (*) for Fisher exact test

Bivariate logistic regression showed that tattoo/scarification, injection drug use, history of blood transfusion, sexual experience, shaving equipment sharing and multiple sexual partners were not risk factors for distribution of HCV prevalence in the studied population (*p* > 0.05). Detail of the seroprevalence of the studied inmates based on the risk factors assessed are represented in Table 3.

**Table 3:** Bivariate logistic regression of some risk behaviors among the studied prison population

Risk factor	No. screened	No. reactive (%)	No. non-reactive (%)		OR	*P* value	CI
*Tattoo/scarification*							
Yes	34	8 (23.5)	26 (76.5)		1.493	0.377	0.613–3.638
No	108	34 (31.5)	74 (68.5)				
*Injection drug use*							
Yes	27	8 (29.6)	19 (70.4)		0.997	0.997	0.398–2.498
No	115	34 (29.6)	81 (70.4)				
*History of blood transfusion*							
Yes	15	5 (33.3)	10 (66.7)		0.822	0.736	0.263–2.570
No	127	37 (29.1)	90 (70.9)				
*Sexual experience*							
Yes	141	42 (29.8)	99 (70.2)		6.8E8	1.000	0.000–0.000
No	1	0 (0.0)	1 (100.0)				
*Shaving equipment sharing*							
Yes	52	11 (21.2)	41 (4.7)		1.958	0.097	0.884–4.337
No	90	31 (34.4)	59 (65.6)				
*Multiple sex partner*							
Yes	99	28 (28.3)	71 (71.7)		1.224	0.608	0.565–2.653
No	43	14 (32.6)	29 (67.4)				
*Previous incarceration*							
Yes	22	8 (36.4)	14 (63.6)		1.445	0.448	0.556–3.756
No	120	34 (28.3)	86 (71.7)				
*Duration in prison*							
≤ 12	85	35 (41.2)	50 (58.8)		1.1E9	0.050	0
13–24	30	4 (13.3)	26 (86.7)		2.4E8		
25–36	18	3 (16.7)	15 (83.3)		3.2E8		
37–48	3	0 (0.0)	3 (100.0)		1.000		
≥ 49	6	0 (0.0)	6 (100.0)				

Table 4 shows the analysis of the interaction of multiple risk factors and seropositivity of HCV among the studied prison inmates. There was no significant (*p* > 0.05) association between seropositivity and the number of risk factors.

**Table 4:** Analysis of the interaction of multiple risk factors and seropositivity of HCV among the studied prison inmates

No. of risk factor	No. Screened	No. reactive (%)	No. non-reactive (%)	β	*p* value	Exp(B)
Nill risk factor	1	0 (0.0)	1 (100.00)	0.000	1.000	1.000
1 risk factors	11	4 (36.4)	7 (63.6)	20.463	1.000	9.231E8
2 risk factors	20	7 (35.0)	13 (65.0)	20.584	1.000	8.699E8
3 risk factors	33	11 (33.3)	22 (66.7)	20.510	1.000	8.077E8
4 risk factors	37	13 (35.1)	24 (64.9)	20.590	1.000	8.750E8
5 risk factors	21	1 (4.8)	20 (95.2)	18.207	1.000	8.077E8
6 risk factors	10	4 (40.0)	6 (60.0)	20.797	1.000	1.076E9
7 risk factors	6	2 (33.3)	4 (66.7)	20.510	1.000	8.077E8
8 risk factors	2	0 (0.0)	2 (100.0)	0.000	1.000	1.000
9 risk factors	1	0 (0.0)	1 (100.0)	0.000	1.000	1.000

## Discussion

The overall prevalence of HCV observed in this study was 29.6% (n = 42/142). The observation is of public health concern as this value is approximately 14 times higher than among the Nigeria general population (2.0%) [31] and 8 times higher than among blood donors (3.6%) in Calabar metropolis [7]. The increased seroprevalence of HCV among inmates compared to the general population in this study supports the previous reports that prisoners represents a high risk group for the transmission of blood borne and sexually transmitted diseases [18, 32, 33]. The prevalence of HCV in this study is higher than previous study in another prison in Nassarawa State, Nigeria which reported 12.3% HCV prevalence among prison inmates [29]. We could not pin point the exact reason for the difference as both studies used the same methods, however, it may be due to variation in prison population as well as variation in risk behaviors among the different prison population [13]. Again, HCV seroprevalence in Nigeria vary based on studied population and geographical region [34]

When compared to international studies, the prevalence of HCV found in our study is lower than that reported in prison studies from Iran (31.5%) [35], Italy (38%) [36], Indonesia (34.3%) [37], Australia (33.3%) [38] and California (34.3%) [39]. Conversely, the prevalence of our study is higher than previous prison studies from Ethiopia (2.6%) [40], Togo (6.0%) [41], Brazil (13.6%) [42], Ghana (19.2%) [32], Ghana (18.7%) [1], England and Wales (7.0%) [43], USA (10.1%) [2], Pakistan (15.2%) [44], Lebanon (3.43%) [45], Spain (22.7%) [46], Germany (8.6%) [47], Mexico (4.9%) [48], France (4.8%) [49] and Scotland (19.0%) [50]. The observed difference in the prevalence of HCV in prisons in the different countries could be due to the type of prison population studied on the basis of significant risk factors such as injection drug use, practice of high risk sexual behaviour, history of imprisonment as well as other high risk behavior prevalent in the studied population [51]. More so, the choice of HCV screening test method could as well influence the variations observed [52].

Prevalence of HCV in this study was observed to be higher among males (31.0%) than females (15.4%). However, the difference was not statistically significant (*p* > 0.05). Puga et al*.* [13]*,* and Adjei and colleagues [1] reported similar trend but a significant higher value in the males than females. Conversely Poulin and colleagues and Vescio et al*.,* reported higher values for females than males [53, 54]. These gender disparities have been attributed to variation in risk factors among both genders [13].

In our study, age was found not to be associated with HCV prevalence among the prison population we studied. This trend is similar to the report of Kebede and colleagues [40]. However, there are contrasting results of increasing age being associated with HCV prevalence [13, 48]. Puga and colleagues attributed the significant association of increasing age and HCV seroprevalence to prolonged exposure to risk factors to the infection.

Duration of incarceration was found to be significantly associated with HCV seroprevalence. Surprisingly, the highest prevalence was observed in those incarcerated < 12 months periods. This points towards the conclusion that majority of the inmates in our study population probably were infected before incarceration. However, contrasting report has linked longer incarceration to higher risk of HCV prevalence [13]. This disparity might be due to the skewed distribution of our studied prison population as majority (59.9%) of our studied prison inmates have been incarcerated less than twelve months. A study should be undertaken which checks for hepatitis C seropositivity at the time of admission of prisoners into the prison and check the same prisoners after a particular time period. This will exactly give the risk of acquiring hepatitis C within prison.

In this study, bivariate logistic regression of the data did not identify injection drug use as risk factor for HCV infection. This is further corroborated by low population of injection drug uses observed in this study. Injection drug use is not popular among Nigerian prisoners when compared to other illicit drug practice [29, 55]. Smoking of cannabis (hemp and marijuana) and cigarette rather than injection are more common substance abuse observed in Nigeria prisoners [27, 55, 56]. However, injection drug has been previously reported as a risk factor in HCV infection in earlier studies in Brazil [13], Mexico [48] and Iran [51]. Furthermore, the low number of participants with these risk factors might have lowered the power of the risk association to be detected.

More so, in this study, tattoo/scarification, history of blood transfusion, sexual experience, shaving equipment sharing, multiple sexual partners and previous incarceration were found not to be associated with HCV prevalence. The low number of participants with known risk factor such as history of blood transfusion might have lowered the power of the risk association to be detected. The realization that a substantial sub-cohort of the studied population might have been infected before incarcerated might have accounted for non association of most of the risk factors assessed in this study.

The recidivism rate of 15.7% among the prisoners observed in this study is a cause of public health concern as these could serve as source of inter and intra-prison transmission of HCV. This proportion of prison inmates shuttling in and out of prison (repeated offenders) is a potential way of spreading HCV to the society. Hence, the need to enact policies that would aid in confinement of the spread of infectious diseases by released prisoners.

As a limitation, we believed that some demographic risk factors may have been underreported owing to fear of punishment, discrimination and stigmatization associated with some social behaviour in developing countries. Furthermore, it is pertinent to note that the result of this study show the percent of participants who have been infected at some time and not just who is currently infected.

## Conclusion

Hepatitis C Virus is highly prevalent among the prison population studied when compared with the general Nigeria population and blood donors. This high seroprevalence of HCV poses a public health concern. We did not find association between HCV seroprevalence and tattoo/scarification, injection drug use, history of blood transfusion, sexual experience, shaving equipment sharing, multiple sex partner and previous incarceration. The result of this study should promote consideration of routine hepatitis C virus antibody screening and behavioral interventions among incarcerated men and women.

## Data Availability

Datasets generated and analysed in this study are available from the corresponding author on request.

## Abbreviations

WHO: World health organization
HCV: Hepatitis C virus
DAA: Direct acting retrovirals
LGA: Local government area
mL: Milliliter
RDT: Rapid diagnostic tests
STI: Sexually transmitted infections

## References

[1] Adjei AA, Armah HB, Gbagbo F, Ampolo WK, Quaye IKE, Hesse IFFA (2007). Corelatiion of hepatitis C virus infection among incarcerated Ghanians: a natural multicentre study. J Med Microbiol.

[2] Alvlarez KJ, Befus M, Herzig CTA, Larson E (2014). prevalence and correlates of hepatitis C virus infection among inmates at two New York State correctional facilities. J Infect Public Health.

[3] Centre for Disease Control and Prevention (US). Guidelines for Viral hepatitis surveillance and case management; 2019. Available at: https://www.cdc.gov/hepatitis/statistics/surveillance-guidelines. Accessed 13 Feb 2019

[4] Strickland UT (2002). Hepatitis C in developing countries. Infect Dis.

[5] Ogar CO, Okafor IM, Akpan PA (2016). Seroprevalence of hepatitis C virus infection among health centre workers in Calabar, Cross River State. Int J Biomed Lab Sci.

[6] World Health Organization (WHO). Global Hepatitis Report 2017. Geneva: World Health Organization. p. 14–41. 2017.

[7] Okoroiwn HU, Okafor IM, Asemota EA, Okpokam DC (2018). Seroprevalence of transfusion-transmissible infectiion (HBC, HCV, Syphilis and HIV) among prospective blood donors in a tertiary health care facility in Calabar, Nigeria; an eleven years evaluation. BMC Public Health.

[8] World Prison Brief. World Prison Population List. Institute for criminal policy research. 2018. p2.

[9] World Prison Brief. Highest to lowest-prison population total. Institute for criminal policy Research. Available at: www.prisonstudies.org/highest-to-lowest/prison-population-total?field_region_taxonomy_tid=15. Accessed 13 Feb 2019.

[10] World Prison Brief. Highest to how- prison population total. Institute for criminal policy Research. Available at:www.prisonstudies.org/highest-to-lowest/prison-population-total?field_region_taxonomy_tid=All. Accessed 13 Feb 2019.

[11] United Nations. 45/11 Basic Principles for the treatment of prisoners. 1990. Available at:https://www.un.org/documents/ga/res/45/a45rlll.htm. Accessed 14 Feb 2019.

[12] Bretschneider W, Elger BS (2014). Expert perspectives on western European Prison Health service: do ageing prisoners receive equivalent care?. J Bioeth Inq.

[13] Puga MAM, Bandeira LM, Pompillio MA, Croda J, de Rezende GR, Dorisbor LFP (2017). prevalence and incidence of HCV Infection among prisoners in Central Brazil. PLoS ONE.

[14] Shin HR, Kim JY, Cao K, Mizokami M, Risch H, Kim SR (2000). Prevalence and risk factors of hepatitis C virus among Koreans in rural area of Korea. Hepatol Res.

[15] Bair RM, Baillargeon JG, Kell PJ (2005). Prevalence and risk factors for hepatitis C virus infection among adolescents in detention. Arch Pediatr Adolesc Med.

[16] Delage G, Infante-Rivard C, Chiiavelta J, Willems B, Pi D, Fast M (1999). Risk factors for acquisition of hepatitis C viral infection in blood donors: result of a case control study. Gastoenterolgy.

[17] Lankaran KB, Ardebili M, Sepehrimanesh M, Nejabat M, Rad MAH, Hosseni SY (2016). Evaluation of hepatitis C virus intrafamilial transmission among families with one index case, a pilot study from Fars province. Iran Gastroenterol Hepatol Bed Bench.

[18] Dufour MC (1999). What is moderate drinking. Alcohol Res Health.

[19] Comfort ML, Ghristead O (2004). The Carceral Limb of the public body: jail inmates, prisoners and infectious disease. AIDS Care.

[20] Liao KF, Lai SW, Chang WL (2006). Screening for viral hepatitis among male non-drug abuse prisoners. Scand J Gastroenterol.

[21] Zampino R, Coppola N, Sagnelli C, Di Caprio G, Sagnelli E (2015). Hepatitis C viral infection and prisoners: epidemiology, outcome and treatment. World J Hepatol..

[22] Larney S, Kopinski H, Beckwith CG, Zaller ND, Jarlais DD, Hagan H (2013). Incidence and prevalence of hepatitis C in prisons and other closed settings; results of a systematic review and meta-analysis. Hepatology.

[23] Weinbaum CM, Sabin KM, Santibanez SS, Hepatitis B, Heptitis C, and HIV in correctional populations: a review of epidemiology and prevention, AIDS. 2005; 19 (suppl 3): S41–6. 10.1097/01.aids.0000192069.95819.aa 16251827

[24] Nwabueze BO (1982). A constitutional history of Nigeria.

[25] Wikipedia, the free encyclopedia, Geography of Cross River State. Available at: https://en.wikipedia.or/wiki/Cross_River_State. Accessed 16 Feb 2018.

[26] Okoroiwu HU, Asemota EA (2019). Blood donors deferral prevalence and causes in a tertiary health care hospital, southern Nigeria. BMC Health Service Res.

[27] National Bureau of statistics. Annual Abstract of Statistics 2011. NBS Federal Republic of Nigeria.

[28] Ekere EF, Useh MF, Okoroiwu HU, Mirabeau TY (2020). Cysteine-cysteine chemokine receptor 5 (CCR5) profile of HIV-infected subjects attending University of Calabar Teaching Hospital, Calabar Southern Nigeria. BMC Infect Dis.

[29] Adoga MP, Banwat EB, Forbi JC, Nimzing L, Pam CR, Gyar SD, Agabi YA, Agwale SM (2009). Human immunonodeficiency virus, hepatitis B virus and hepatitis C virus: sero-prevalence, co-infection and risk factors among prison inmates in Nasarawa State, Nigeria. J Infect Dev Ctries.

[30] Naing NN (2003). Determination of sample size. Malays J Med Sci.

[31] World Health Organization (WHO). Nigeria Employs numerous strategies to create awareness on viral hepatitis nationwide. Available at:https://www.afro.who.int/news/nigeria-employs-numerous-strategies-create-awareness-viral-hepatitis-nationwide. Accessed 14 Feb 2019.

[32] Adjei AA, Armah HB, Gbagbo F, Ampolo WK, Quaye IKE, Hesse IFA (2006). Prevalence of human immunodeficiency virus, hepatitis B Virus, hepatitis C virus and syphilis among prison inmates and officers at Nsawam and Accra, Ghana. J Med Microbial.

[33] Alizadeh AHM, Alavian SM, Jafari K, Yazdi N (2005). prevalence of hepatitis C virus infection and its related risk factors in drugs abuser prisoners in Hamedan Iran. World J Gastroenterol.

[34] Onyekwere C A, O Ogbera A, Olusola Dada A, O Adeleye O, O Dosunmu A, et al. Hepatitis C Virus (HCV) prevalence in special populations and associated risk factors: a report from a Tertiary Hospital, Hepat Mon. 2016; 16(5):e35532. 10.5812/hepatmon.35532 PMC4908612 27313634

[35] Alavian SM, Fallahian F (2009). Epidemiology of hepatitis C in Iran and the world. Shiraz E Med J.

[36] Almasio PL, Babudie S, Barbarini G, Brunetto Conte D, Dentico P (2011). Recommendation for the prevention, diagnosis and treatment of chronic hepatitis B and C in special population groups (migrants, intravenous drug users and prison inmates). Dig Liver Dis..

[37] Prasetgo AA, Dirgahyu P, San Y, Hudigono H, Kageyama S (2013). Molecular epidemiology of HIV, HCV and HTLV-1/2 in drug user inmates in central Javan prison, Indonesia. J Infect Dev Ctries.

[38] Reekie JM, Levy MH, Richards AH, Wake CJ, Jiddall DA, Beasley HM (2014). Trends in HIV, hepatitis B and hepatitis C prevalence among Australia prisoners, 2004, 2007, 2010. Med J Aust.

[39] Fox RK, Currie SL, Evans J, Wright TL, Tobler L, Phelp B (2005). Hepatitis C virus infection among prisoners in the California state correctional system. Clin Infect Dis.

[40] Kebede W, Abdissa A, Seid Y, Mekonnen Z (2017). Seroprevalence and risk factors of hepatitis B, hepatitis C and HIVinfectiions among prisoners in Jimma Town, Southwest Ethiopia. Asian Pac J Trop Dis.

[41] Jaquet A, Wandeler G, Tine J, Dagnra CA, Ahia A, Patassi A (2016). HIV infection, viral hepatitis and liver fibrosis among prison inmates in West Africa. BMC Infect Dis.

[42] Magri MC, Ibrahim KY, Pinto WP, Franca FS, Bernardo WM, Tengan FM (2015). Prevalence of hepatitis C virus in Brazil’s inmate population: a systematic review. Rev Saude Publica.

[43] Weild AR, Gill ON, Bennett D, Livingstone SJ, Parry JV, Curran L (2000). Prevalence of HIV, hepatitis B, and hepatitis c antibodies in prisoners in England and Wales: a national survey. Commun Dis Public Health.

[44] Kazi AM, Shah SA, Jenkins CA, Shepherd BE Vermund SH. Risk factors and prevalence of tuberculosis, human immunodeficiency virus, syphilis, hepatitis B virus, and hepatitis C virus among prisoners in Pakistan. Int J Infect Dis. 2010; 14 (suppl. 3): e60–6. 10.1016/j.ijid.2009.11.012 PMC2905608 20189863

[45] Mahfoud Z, Kassak K, Kreidieh K, Shamra S, Ramia S (2010). prevalence of antiboides to human immunodeficiency virus (HIV), hepatitis B, and hepatitis C and risk factors in prisoners in Lebanon. J Infect Dev Ctries..

[46] Saiz de la Hoya P, Marco A, Garcia-Gueriero J, Rivera A. Hepatitis B and C prevalence in Spanish prisons. Eur J Clin Microbial Infect Dis. 2011; 30: 857–862. 10.1007/s10096-011-1166-5 21274586

[47] Meyer MF, Wedemeyer H, Monazahian M, Dresman J, Manns MP (2007). Prevalence of hepatitis C in a German prison for young men in relation to country of birth. Epidemiol Infect.

[48] Belaunzaran-Zamudio PF, Monsqueda –Gomez JL, Macias – Hernandez A, Sierra-Mandero JG, Ahmed S, Beyrer C. Risk factors for prevalent hepatitis C virus—infection among inmates in a state prison system Mexico. PLoS ONE. 2017; 12 (6): e0179931. 10.1371/journal.pone.0179931 PMC5487058 28654650

[49] Semaille C, Le Strat Y, Chiron E, Chemlal K, Valantin MA, Serre P, et al. prevalence of human immunodeficiency virus and hepatitis C virus among prison inmates in 2010: a challenge of public health policy. Euro Surveillance. 2013;18(28): Pii: 20524. 10.2807/1560-7917.es2013.18.28.20524 23870097

[50] Taylor a, Munro A, Allen E, Dunleavy K, Cameroon S, Miller L, et al. Law incidence of hepatitis C virus among prisons in Scotland. Addiction. 2013; 108(7): 1296 -304. 10.1111/add.12107 23297816

[51] Behzadifar M, Gorji HA, Rezapour A, Bragazzi NL (2018). Prevalence of hepatitis C virus infection among prisoners in Iran: a systematic review and meta-analysis. Harm Reduction J..

[52] Petruzziello A, Marigliano S, Loquercio G, Cozzolino A, Cacciapuoti C (2016). Global epidemiology of hepatitis C virus infection: An up-date of the distribution and circulation of hepatitis C virus genotypes. World J Gastroenterol.

[53] Poulin C, Alary M, Lambert G, Godin G, Landry S, Gagnon H (2007). prevalence of HIV and hepatitis C virus infections among inmates of quebec provincial prisons. CMAJ.

[54] Vescio MF, Lango B, Babudieri S, Starnini G, Carbonara S, Rezza G (2008). Correlates of hepatitis and community Health. J Epidemiol Community Health.

[55] Adesanya A, Ohaeri JU, Ogunlesi AO, Adamson TA, Odejide OA (1997). Psychoactive substance abuse among inmates of Nigerian prison. Drug Alcohol Depend.

[56] Armiya’u AY, Perez A. Demographic factors, forensic profile, substance abuse and crime in violent offenders at maximum security prison in North Central Nigeria. J Forensic Sci Criminal Inves. 2016; 1(2):001–010.

